# Effect of fungal species on thermal conductivity and chemical composition in mycelium-based insulation

**DOI:** 10.1038/s41598-025-33828-4

**Published:** 2026-01-02

**Authors:** Joni Wildman, Daniel Henk, Pete Walker, Andrew Shea

**Affiliations:** 1 Department of Civil Engineering and Architecture, University of Bath, Bath, UK; 2 Milner Centre for Evolution, University of Bath, Bath, UK

**Keywords:** Bio-based insulation, Sustainable construction, Circular economy, Mycelium-based composites, Civil engineering, Engineering, Materials science, Applied microbiology, Fungi

## Abstract

The construction industry is a major contributor to carbon emissions and landfill waste, leading to the need for sustainable insulation alternatives. Mycelium-based composites (MBCs) have emerged as promising alternatives; they are bio based materials derived from the growth of fungal mycelium on lignocellulosic substrates. They offer low embodied carbon, waste utilisation, and low thermal conductivity ($\lambda$). However, the influence of fungal species on MBC properties (e.g. $\lambda$) remains underexplored. This study investigates the extent to which fungal species selection affects MBC thermal performance and chemical composition. MBCs were successfully produced using 18 different fungal strains (17 species) on a hemp-shiv substrate with aesthetic and textural differences observed between specimens. $\lambda$ was measured using a Heat Flow Meter (ISO 8301, EN 12667, and ASTM C518). Across the different species, all MBCs measured in this study exhibited good insulation properties, with thermal conductivity values ranging from $0.0376 \pm 0.0006$ W/m·K (*Pholiota adiposa*) to $0.0451 \pm 0.001$ W/m·K (*Lentinus tigrinus*), with statistically significant differences. Fourier Transform Infrared Spectroscopy with Attenuated Total Reflectance (FTIR-ATR) and multivariate analysis (PCA and LDA) were used to assess chemical differences. While the multivariate analysis revealed species-specific differences in the FTIR spectra of the specimens, no significant correlation was found between spectral features and $\lambda$, suggesting that physical structure and density play a more dominant role in heat transfer properties. This study represents a large interspecies comparison of the thermal properties of MBCs, demonstrating that a range of fungal species can produce effective insulation materials. It also highlights the effectiveness of multivariate analysis in comparing FTIR spectra of bio-based materials, offering a framework for characterising chemical modifications in sustainable insulation materials. By improving understanding of species selection and its impact on thermal performance, this research contributes to advancing MBCs as effective insulation for sustainable construction.

## Introduction

The construction industry is responsible for 36% of CO$_2$ emissions worldwide and 45–65% of the waste in landfill^1,2^. Operational carbon accounts for 73% of construction industry emissions and is due to the energy consumption of a building through its lifetime, which is largely due to heating and cooling^1,3^. Effective insulation materials are essential to reduce this energy demand^4^. Insulation materials reduce the transfer of heat between spaces at different temperatures and their thermal performance can be assessed through their thermal conductivity ($\lambda$) which quantifies the heat transferred according to Fourier’s law of heat conduction: $q = {-}\lambda \nabla T$^5^. Insulation materials can be considered effective if $\lambda$ is below 0.1 W/m·K^6^. However in addition to operational carbon demands, 27% of construction industry emissions are from the manufacture of building materials; traditional insulation materials, such as expanded polystyrene, can have a high embodied carbon- emissions associated with their manufacture^3,7^. These materials are also often non-degradable, non-recyclable, energy-intensive to manufacture, and deplete finite resources. Recyclable, low-embodied energy, and effective alternative insulation materials are therefore urgently needed that align with the goals of a more circular and lower emission construction industry^8,9^. Mycelium-based composites (MBCs) have emerged as promising alternatives to unsustainable insulation materials^10^. MBCs are formed when fungal mycelium is grown on a substrate. Mycelium is the vegetative part of a fungus, consisting of branching hyphal cells that form a network of thread-like filaments^11^. The primary substrates used to form MBCs are lignocellulosic materials, which consist of lignin, cellulose, and hemicelluloses^12^. MBCs can not only utilise waste streams (e.g. agricultural and paper waste), but have also exhibited low embodied carbon, gaining them attention as a sustainable option^13^. Furthermore, MBCs have demonstrated effective insulation properties, with thermal conductivity values comparable to those of traditional materials, with multiple studies measuring MBCs with values below 0.1 W/m·K as Wildman et al. recently reviewed^14^. $\lambda$ is a critical parameter for assessing the performance of insulation materials by quantifying the heat transfer through the material, and is thus important for evaluating the efficacy of MBCs as insulation^14^. The fungal species used to produce MBCs is a key factor influencing their thermal conductivity, as well as other attributes such as aesthetic properties, manufacturability, structural integrity, density, and the formation of a fungal skin on the material^15–17^. Selecting the appropriate fungal species has been identified as crucial when optimising MBCs for insulation applications, and a variable that necessitates further exploration^14,18^.

Various studies have demonstrated the potential of several fungal species for MBC production, yet comprehensive evaluations of interspecies variation in thermal performance remain limited^12^. To date, 14 species have been used to form MBCs that have been thermally characterised using thermal conductivity, with up to 3 species being compared in a single study^14^. Verhelst et al. assessed the thermal performance of nine fungal species by measuring heat transfer through mycelium composites and determining their equivalent extruded polystyrene (XPS) thickness^19^. They found significant variation between species, but comparisons with other studies are challenging due to the lack of a standardised measurement method or direct thermal conductivity values^19^. Testing a wider range of fungal species can help with optimisation, as well as providing information on which substrate species combinations are viable for effective MBC production, such that species can be selected based on availability and desirable properties from a diverse range of candidates^20^. Beyond this, further investigation is needed to better understand why different fungal species produce MBCs with varying properties and to what extent these differences can be attributed to species selection. Exploring these factors contributes to more informed material development and optimisation. Previous research has explored the influence of fungal species on MBC variability. Jones et al. investigated the relationship between hyphal branching and growth rate, finding that a higher branching density was associated with a slower extension rate, which in turn affected material properties^17^. However, factors such as pathogenicity, taxonomic classification, and fungal associations were not reliable indicators of growth performance^17^. Haneef et al. examined differences in hyphal morphology between *Ganoderma lucidium* and *Pleurotus ostreatus* and reported that the different species change their hyphal structure differently depending on the substrate^21^. They also identified chemical variations between the species, with *Ganoderma lucidium* exhibiting a higher lipid content, as well as differences in chitin composition. These findings were used to explain mechanical performance, with the greater flexibility of *Ganoderma lucidium* derived MBCs attributed to their higher protein and lipid content, which may act as natural plasticisers^21^. The way fungi interact with and modify a substrate influences key properties of MBCs, including porosity, density, and chemical composition, all of which could potentially impact heat transfer characteristics and thermal conductivity^22^. Fungal growth is also affected by environmental factors such as oxygen, moisture, substrate composition, and temperature, which can therefore influence MBC formation^11,23^. For example, limiting oxygen availability can restrict microbial and fungal activity, and maintaining low moisture content is an effective way to suppress fungal growth in lignocellulosic materials^24^. Thermal modification of wood, which can change the chemical, physical and mechanical properties of the material, has been shown to alter its susceptibility to certain decay fungi^24^. Temperature more broadly affects mycelial growth kinetics, with optimal ranges typically between 20–30 $^{\circ }$C^25^. Techniques like Fourier Transform Infrared Spectroscopy (FTIR) provide a method for analysing how different fungal species alter the substrate, offering insight into the biochemical processes underlying material variation^26^. However, the extent to which these species-specific chemical differences are measurable and significant, and whether they correlate meaningfully with thermal performance, remains an open question. Addressing this gap could help clarify the role of fungal species in determining MBC properties and reduce uncertainty regarding their impact on thermal performance.

Overall this study aims to investigate the extent to which fungal species selection affects the thermal conductivity of MBCs, providing insights that can aid in optimising their performance and their viability as an effective and sustainable insulation alternative. The aims of the study are as follows:

- To produce MBCs using 18 different fungal strains (17 different species) in order to conduct a large inter-species comparison of MBC material properties and provide information of viable species-substrate choices
- To quantify how the choice of fungal species influences the thermal conductivity of MBCs grown on hemp-shiv
- To assess how MBC formation affects the chemical properties of hemp-shiv, and to determine whether MBCs produced with different fungal species can be categorised based on the FTIR spectra of the MBC using multivariate analysis
- Assess whether variations in thermal conductivity correlate with differences, identified using the multivariate analysis, in the FTIR spectra of the different MBCs

## Materials and methods

### Specimen preparation

#### Fungal species

17 different fungal species were used in this study, with 2 different cultures used for *Trametes versicolor* (one commercial and one wild). These fungal species are considered selective white rot fungi meaning that they preferentially degrade lignin while only partially breaking down cellulose and hemicelluloses, which is important for MBC production, as the retention of cellulose and hemicelluloses helps maintain structural integrity^23^. Table 1 shows the species used, their origin, and the code assigned to that species for this study.

All species except TV2 were obtained in liquid culture from the supplier. The identity of the species was verified based on sequencing of the ITS4 region. These cultures were then used to inoculate MEYA (50 g/L MEA, 2 g/L yeast extract) plates and healthy mycelium was allowed to colonise the plates. Once the plates were colonised they were used to inoculate grain. Sterilised and hydrated food-grade brown rice was used to produce grain spawn. 1 cm$^2$ squares of the healthy mycelium growing on MEA plates were used to inoculate sterile rice and allowed to fully colonise the rice. Once fully colonised with healthy mycelium (assessed visually with the mycelium covering the grain), this grain spawn was combined with the substrate in subsequent steps of the production process.

**Table 1:** Species used to form the Mycelium-Based Composites in this study and their assigned code.

Species	Code	Source
*Agrocybe aegerita*	AA	Mycopunks
*Ganoderma applanatum*	GA	KingdomLCs
*Ganoderma curtisii*	GC	KingdomLCs
*Ganoderma lingzhi*	GLI	KingdomLCs
*Ganoderma lucidium*	GL	KingdomLCs
*Ganoderma multipileum*	GM	KingdomLCs
*Ganoderma neo-japonecium*	GNJ	KingdomLCs
*Ganoderma oregonenese*	GO	KingdomLCs
*Laetiporus sulphureus*	LS	Gourmet Woodland Mushrooms
*Lentinus tigrinus*	LT	KingdomLCs
*Pholiota adiposa*	PA	Mycopunks
*Pleurotus ostreatus v. columbinus*	PC	Gourmet Woodland Mushrooms
*Pleurotus citrinopileatus*	PCI	KingdomLCs
*Pleurotus djamor*	PD	KingdomLCs
*Pleurotus eryngii*	PE	Mycopunks
*Pleurotus nebrodensis*	PN	LincsMushroomShop
*Trametes versicolor*	TV	Beacon Hill
*Trametes versicolor*	TV2	Cloned from specimen (Bath, UK)

#### Substrate preparation

Hemp chips (hemp-shiv) were acquired from ProRep (www.pro-rep.co.uk). The hemp chips were untreated with average chip size 10 mm x 2 mm. The hemp-shiv was soaked in water for 24 h before being drained and packed in propylene autoclave bags. Then the hemp was sterilised in an autoclave (121 $^{\circ }$C and 15 psi for 30 min) and allowed to cool before use in subsequent stages of specimen preparation.

#### Composite production

**Fig. 1:**
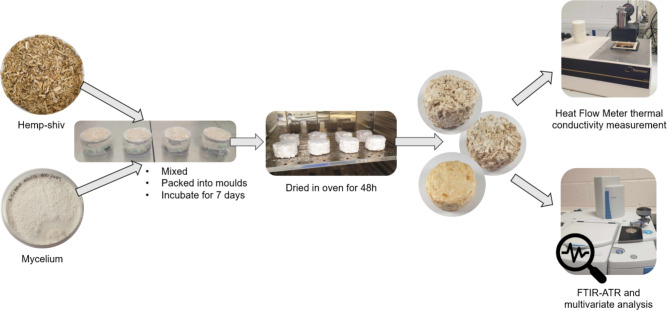
Diagram showing the stages of MBC production and analysis in this study.

Figure 1 shows a diagram of the composite production process. In a biosafety cabinet the substrate and grain spawn were combined in a ratio of 10:1 with the grain spawn broken up into mycelium-colonised individual grains. A large bowl and spoon, cleaned with 70% isopropanol, were used to thourally mix the spawn and substrate. The mixture was then packed into cylindrical moulds (height 5 cm, diameter 8 cm) cleaned with 70% isopropanol. Once the moulds were filled they were covered with a sheet of parafilm secured with tape to allow for air exchange whilst minimising contamination during incubation. The samples were then incubated at 23 $^{\circ }$C until sufficient colonisation and structural integrity had been achieved (determined by full mycelium coverage on external surface and a composite that could maintain structure in the absence of the mould). This took between 3 and 7 days depending on the species. In a biosafety cabinet the composites were then transferred to larger pots cleaned with 70% isopropanol and these pots were again covered with a sheet of parafilm secured with tape. These were then incubated at 23 $^{\circ }$Cfor a further 3 days. This transfer was required to ensure even coverage across all surfaces of the composite and allowing for additional air exchange, by using a larger mould, which promotes fungal growth. The composites were then removed from the pots and weighed. They were then dried in an oven at 50 $^{\circ }$C for 48 h. The composites were weighed after 40 h and after 48 h to ensure that no further mass was lost upon 8 h additional drying thus the composites were not retaining moisture.

For each fungal species/culture, four independent biological replicates were produced, each using a different grain spawn batch to inoculate the hemp-shiv and produce separate MBC specimens. The process was repeated for each species/culture to produce 72 composites in total. Final specimen densities were calculated, and macroscopic images were taken of all specimens (Table 2). After thermal conductivity measurements of each specimen, specimens were cross sectioned in order to confirm that mycelial colonisation had worked throughout the bulk of the substrate (confirmed visual examination of mycelial growth throughout and from the material holding intact when sectioned) and so that the bulk of the specimen could be sampled for FTIR-ATR analysis.

### Thermal characterisation

The thermal conductivity of each specimen was determined using a Heat Flow Meter. Five thermal analysis tests were performed on each of the 76 specimens (380 tests in total) using a Thermtest HFM-25. Each specimen was stored in the laboratory at room conditions for 1 week (approx. 20 $^{\circ }$C and 50% RH) prior to testing. Specimen density was also calculated according to ASTM C303 prior to testing^27^. The test was conducted at a mean temperature of 20 $^{\circ }$C with a 20 $^{\circ }$C temperature difference between the plates.

### FTIR-ATR

This section describes the methodology used to analyse the chemical differences between MBCs produced with different fungal species using Fourier Transform Infrared Spectroscopy with Attenuated Total Reflectance (FTIR-ATR). The experimental procedure for specimen preparation and data acquisition is outlined, followed by an explanation of the multivariate statistical techniques used to interpret spectral variations. Principal Component Analysis (PCA), Linear Discriminant Analysis (LDA), and clustering analysis were used to explore patterns in the spectral data and distinguish differences between specimens. PCA was chosen to reduce the dimensionality of the FTIR spectral data while retaining the most significant variations, allowing for the identification of key wavenumbers that differentiate specimens^28^. LDA was chosen to maximize class separation, ensuring that differences between MBCs made using different fungal species are emphasised based on their spectral profiles^29^. Cluster analysis was used to identify natural groupings within the dataset to assess whether MBCs made with the same species can be grouped based on their FTIR spectra.

FTIR-ATR can be a useful tool for probing the chemical differences between MBCs formed using different fungal species^30^, being an analytical technique used to identify functional groups in materials by producing an infrared absorption spectrum from which chemical composition can be inferred^31^. In this study, FTIR is utilised to measure the chemical changes in a hemp-shiv substrate decomposed by the different selective white-rot fungal species during MBC formation. FTIR is well-suited for comparing spectra between samples within the same experimental design^32^, however even a single change in substrate composition can result in changes across multiple regions of the FTIR profile. In this context, multivariate analysis is a valuable tool for identifying patterns of substrate digestion by different fungal species^33^. Therefore the FTIR data acquired in this study is used to establish multivariate models to identify key wavelengths related to chemical differences in MBCs made using different fungal species.

To acquire the FTIR data, a scalpel was used to cut a piece of each MBC specimen from the interior of the MBC. For each specimen, 2 pieces were sampled taken from different locations: one from the centre and one from an intermediate position between the centre and the outer surface. The cut piece of the specimen was then ground in a pestle and mortar to a coarse powder and transferred to a vial. Hemp-shives were prepared in the same way. The prepared samples were tested with the FTIR-ATR. FTIR-ATR analyses were performed using a Perkin Elmer Frontier FTIR spectrometer, equipped with a diamond ATR head. Background spectra was taken prior to measurements being taken for each set of species samples. Overall, for each group of MBCs made with a different species 8 scans/spectrum were acquired (2 scans/spectra per specimen corresponding to each sampled region) in the 4000-650 cm$^{-1}$ range with a resolution of 4 cm$^{-1}$.

OMNIC paradigm was used to process each spectra. The spectra were autobaseline corrected and normalised before subsequent analysis.

Multivariate analysis was performed using principal component analysis (PCA), linear determinant analysis (LDA), and clustering analysis. In PCA, the data is linearly transformed onto a new coordinate system such that the directions (principal components (PCs)) capturing the largest variation in the data can be identified. The central objective of this technique is to identify sources of data variation, extracting information about the data set from the number of independent PCs that best describe the information, reducing dimensionality without losing relevant information in the data set^28^.

Like PCA, LDA seeks to find linear combinations of variables which best explain the data. LDA explicitly attempts to model the difference between distinct classes of data. PCA, in contrast, does not take into account any difference in class, and factor analysis builds the feature combinations based on differences rather than similarities. LDA can be used in this study as each specimen is assigned to a different class (group) based on the species used to make the composite^29^.

Normalised values of % reflectance at key wavenumbers (as identified using PCA and LDA and measured using FTIR-ATR) were compared between groups of specimens made using different species. For this ANOVA followed by post hoc Tukey’s test was used where comparisons were made at the significance level of 0.05.

Cluster analysis is another multivariate method that groups a set of objects in such a way that objects in the same group (or cluster) are more similar to each other than to those in other groups. This method can be particularly useful in identifying natural groupings in the data, which may correspond to specific degradation patterns or species-substrate interactions^34^. The cluster analysis was conducted on the reduced dimension data set from the PCA and LDA using K-means clustering to identify groupings within the data plotted on the identified PCs and LDs. Additionally, adjusted rand index (ARI) and normalised mutual information (NMI) metrics were calculated to determine if the data points MBCs made using the same species significantly cluster.

The multivariate analysis was performed using Python with the scikit-learn library and statistical analysis was performed using Python with the SciPy.Stats library. PCA and LDA was conducted on the normalised spectra of the 144 samples between the wavelengths 850cm$^{-1}$ to 1750cm$^{-1}$.

### Analysis of thermal conductivity and FTIR spectra

To compare the differences in thermal conductivity measurements made for MBCs made using different species, analysis of variance (ANOVA) followed by post hoc Tukey’s test was used and comparisons were made at the significance level of 0.05. The correlation between the thermal conductivity measurements and the normalised values of % reflectance at key wavenumbers was determined using Pearson’s correlation coefficient and assessed at the 0.05 significance level.

## Results

### Composites

Composites were successfully produced using the 18 different fungal strains, demonstrating good repeatability between replicates, uniformity within each specimen, and good structural integrity. Images of the specimens produced are shown in Table 2, with aesthetic variations between specimens observed, including differences in colour, texture, and the formation of a fungal-skin layer. These results show that the fungal species can be combined with hemp-shiv substrates to form viable MBCs, and that species selection influences the composite morphology.

**Table 2:**
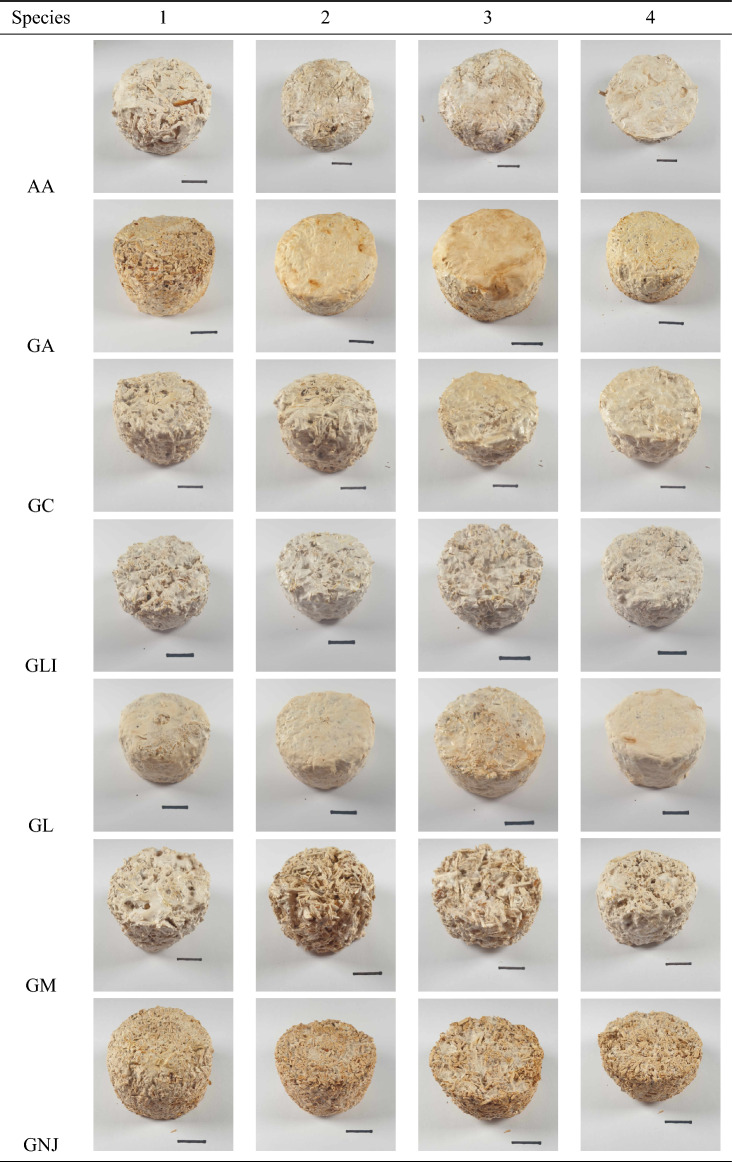

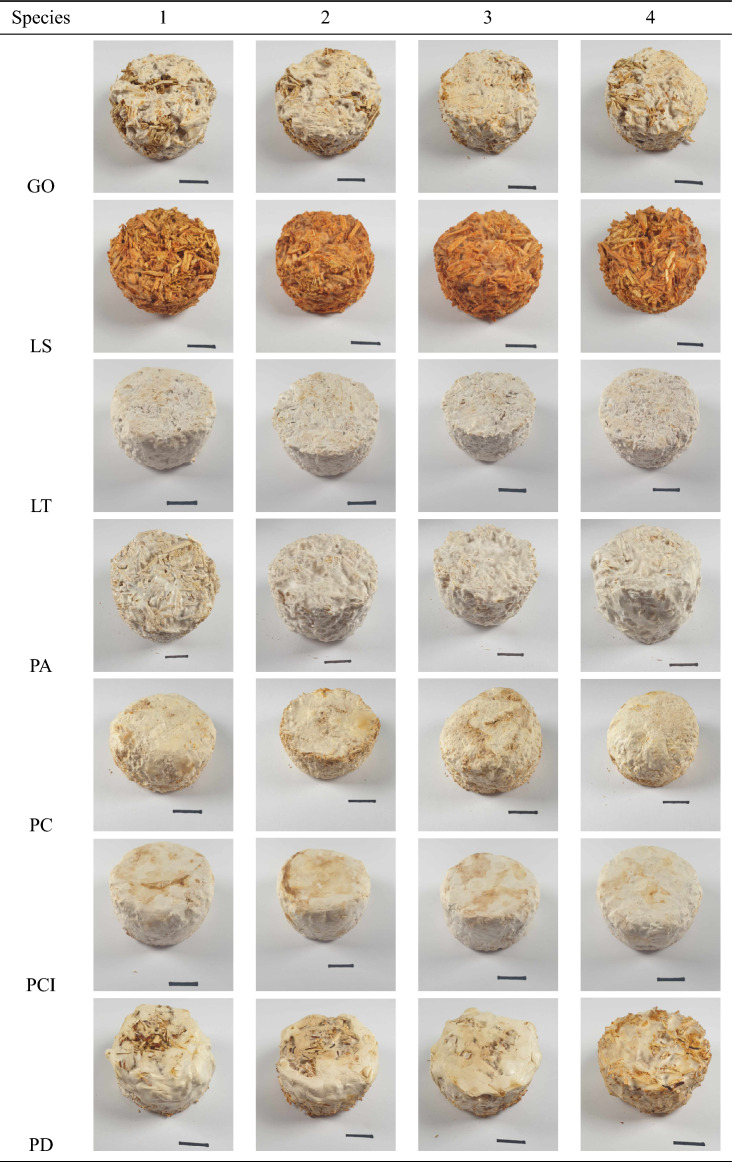

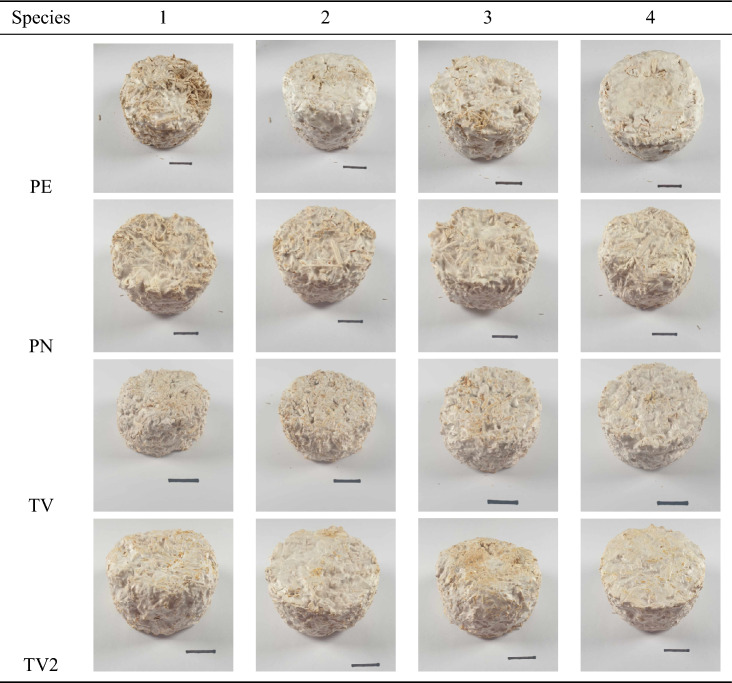
Mycelium-based composites produced using 18 different fungal strains (17 different species) with a hemp-shiv substrate. Scale bar 2cm.

### Thermal characterisation

**Fig. 2:**
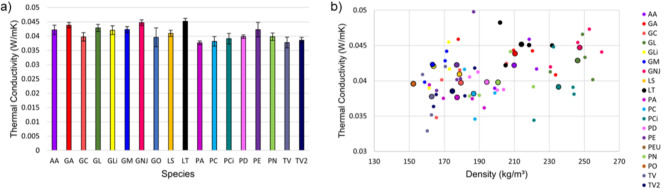
(**a**) Steady-state measurement of thermal conductivity of hemp-based MBCs made with different fungal species. (**b**) Steady-state measurement of thermal conductivity of hemp-based MBCs made with different fungal species as a function of their density. Small points are averages of individual MBCs and large points are averages of the biological repeats from that species.

For the 76 MBC specimens, the mean thermal conductivity measured was $\lambda = 0.0409 \pm 0.0004$ W/m·K. Fig. 2 a) shows the measured values of $\lambda$ for the different species. Based on the statistical analysis conducted on these results, a one-way ANOVA test revealed a statistically significant difference between groups ($p = 0.03 < 0.05$), indicating that $\lambda$ varies among the MBCs made with different species. The lowest value of $\lambda$ was for the MBCs made using *Pholiota adiposa* with $\lambda = 0.0376 \pm 0.0006$ W/m·K and the highest was for those made using *Lentinus tigrinus* with $\lambda = 0.0451 \pm 0.001$ W/m·K. Fig. 2 shows value of $\lambda$ measured for the specimens as a function of density ($\rho$). Pearson’s correlation analysis reveals a moderate positive correlation between $\lambda$ and $\rho$ ($r = 0.408, p = 0.00083 < 0.05$). This indicates that higher $\rho$ is associated with higher $\lambda$, which is statistically significant in the data.

### FTIR-ATR

**Fig. 3:**
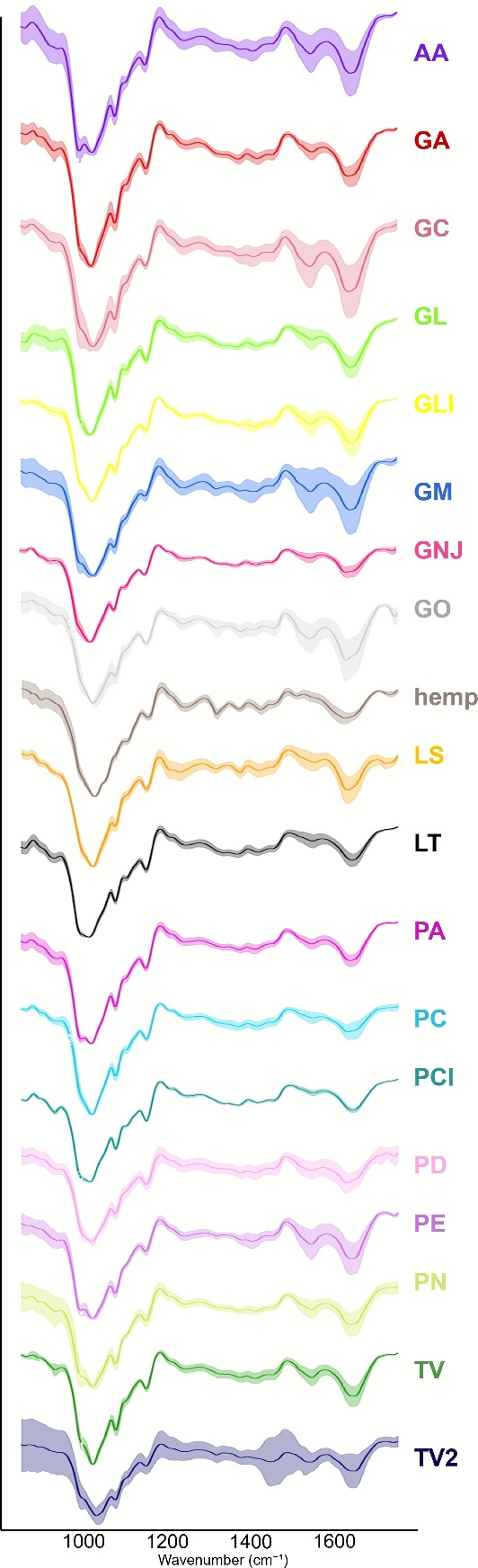
Average FTIR-ATR spectra for the specimen used in this study. Spectra have been baseline corrected and normalised, and standard deviation of spectra for each group is shown.

Figure 3 shows the average FTIR-ATR spectra for the composites made with each d-different species and hemp, along with the standard deviations of spectra. Table 3 shows the prominent peaks identified for the mycelium based composites and hemp, along with the wavenumber assignment. Comparing the peaks identified in hemp and the MBCs we see there are small shifts in many of the wavenumbers such as 3285 cm$^{-1}$, 2918 cm$^{-1}$, 1636 cm$^{-1}$, 1424 cm$^{-1}$, 1370 cm$^{-1}$ and 1155 cm$^{-1}$ suggesting that many of the key functional groups in hemp are retained with slight modifications in the chemical composition of the hemp due to degradation by the fungi. Some wavenumbers are present in one but not the other, such as 1729 cm$^{-1}$ and 1075 cm$^{-1}$, suggesting there are changes in polysaccharide content due to fungal degradation. The peak at 1365 cm$^{-1}$ in the MBCs could be assigned to the chitin in the fungal cell wall, however, the peak at 1370 cm$^{-1}$ in the hemp is likely to be of cellulose and hemicelluloses origin and this could have also contributed to the peak in the MBCs.

**Table 3:** Identified peaks for MBCs and hemp-shiv with peak assignments.

Wavenumber (cm$^{-1}$)	Assignment	Ref.
Mycelium-based composites		
3280 cm	Lignin, cellulose and hemicelluloses: OH intramolecular and intermolecular stretching	^35,36^
2921	lignin, cellulose and hemicelluloses: symmetric methyl and methylene stretching	^35,36^
1640	Lignin and cellulose: absorbed O-H	^37,38^
1535	Lignin: C=C stretching of aromatic ring	^37–39^
1415	Lignin: C=O stretching and aromatic skeltal vibration. Carbohydrates: CH and OH bending	^36,40–42^
1365	Chitin: CH$_2$wagging	^38,43^
1245	Polysaccharides: C-O stretch and O-H in plane	^35,44^
1149	Cellulose: C-O-C asymmetric stretching	^35,36^
1075	Polysaccharides: C–C stretching ring	^45,46^
1015	Cellulose: C=O stretch	^37–39^
930	hemicelluloses and polysaccharides: Ring vibration	^46,47^
860	Mannose-containing polysaccharide: C1–H bending	^46^
Hemp		
3285	Lignin, cellulose and hemicelluloses: OH intramolecular and intermolecular stretching	^35,36^
2918	Cellulose, hemi cellulose: CH stretcing	^48^
1729	hemicelluloses: C=O unconjugated stretching acetyl groups	^48^
1636	Lignin and cellulose: absorbed O-H	^37,38^
1424	Carbohydrates: CH and OH bending	^36^
1370	Cellulose and hemicelluloses: CH bending	^35,36,49^
1318	Cellulose: CH$_2$wagging	^35,36,49^
1245	Polysaccharides: C-O stretch and O-H in plane	^35,44^
1155	Cellulose: C-O-C asymmetric stretching	^35,36^
1100	Cellulose and hemicelluloses: C-O-C stretching. Lignin: aromatic C-H in plane deformation	^35,36,49^
1026	Holocellulose and lignin: C-O stretching	^35,50^
900	Polysaccharides: beta-glycosidic bonds symmetrical ring stretching mode	^48^
875	Mannose-containing polysaccharides: C1–H bending	^46^

### PCA

**Fig. 4:**
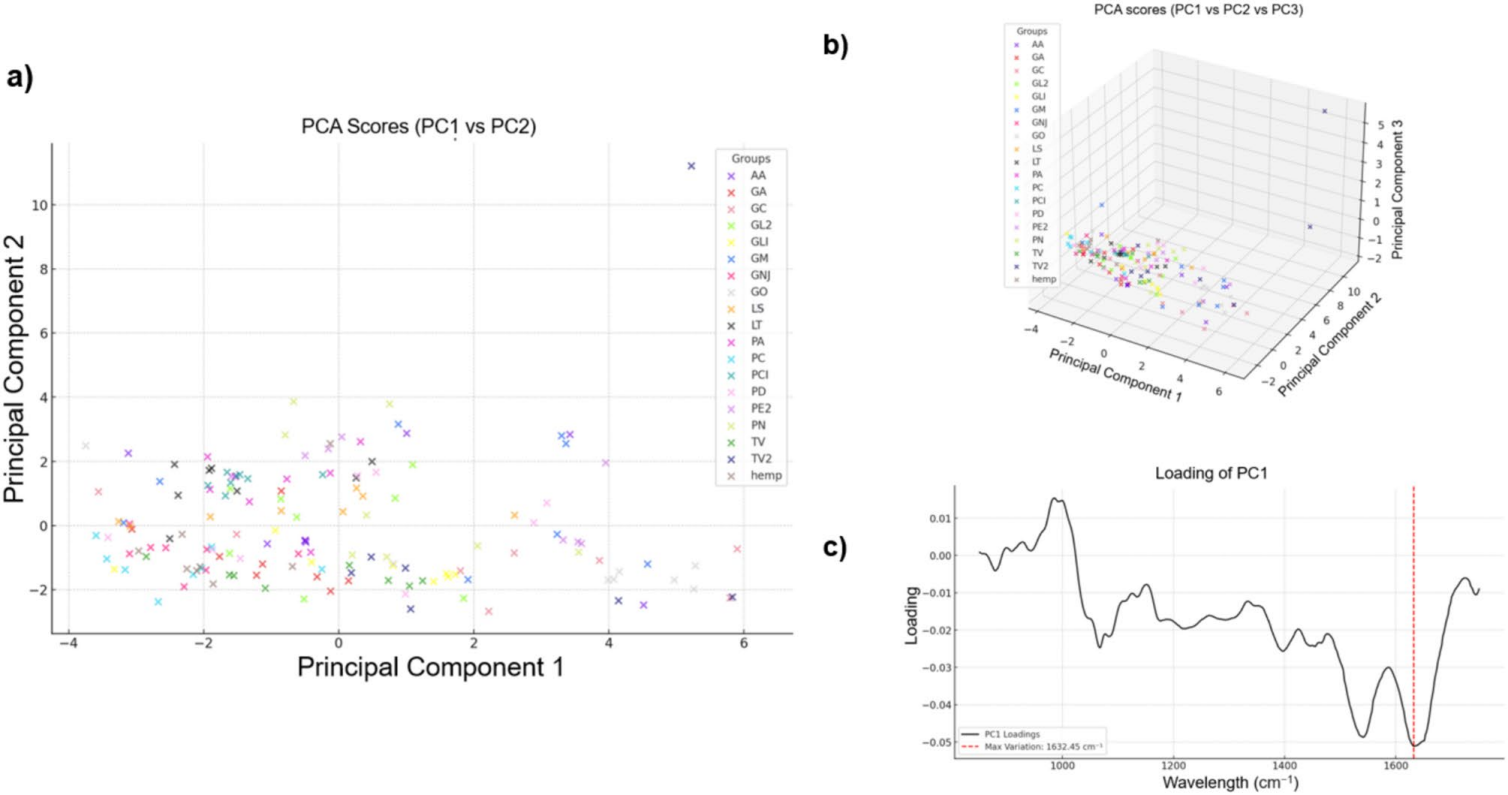
PCA of the FTIR spectra (850–1750 cm$^{-1}$ region) of 72 MBC samples and 8 hemp-shiv samples. (**a**) score plot of PC1 (50.57%) vs PC2 (30.46%), (**b**) score plot of PC1 vs PC2 vs PC3 (5.65%), (**c**) loading profile of PC1.

Figure 4 a) shows the scores of the PCA plotted in the plane of principal component 1 (PC1) and principal component 2 (PC2) where PC1 and PC2 explain approximately 50.57% and 30.46% of the variance in the FTIR-ATR spectral data respectively. Fig. 4 b) show the scores of the PCA plotted with PC3 as a third dimension, of which accounted for an additional 5.65% such that PC1, PC2 and PC3 together account for of 86.67% of the variance in the data. Fig. 4 c) shows the loading plot for PC1 where The loading plot gives information on which wavenumbers mostly contributed to the separation of MBC and hemp samples according the PC1 values. The wavelength with the greatest variation between samples according to the PC1 loading plot was 1632 cm$^{-1}$. Peaks were observed in the spectra of MBCs and hemp at 1640 cm$^{-1}$ and therefore this wavenumber was chosen to calculate average peak ratios to quantify the variation between the specimen.

### LDA

**Fig. 5:**
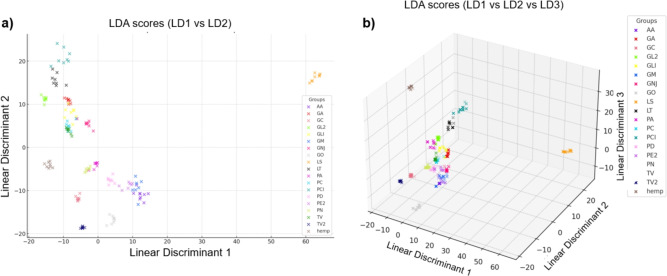
LDA of the FTIR spectra (850-1750cm$^{-1}$ region) of 72 MBC samples and 8 hemp-shiv samples. (**a**) score plot of LD1 (36.62%) vs LD2 (17.09%), (**b**) score plot of LD1 vs LD2 vs LD3 (13.70%).

Figure 5 a) shows the scores of the LDA plotted in the plane of linear discriminant 1 (LD1) and linear discriminant 2 (LD2) where LD1 and LD2 explain approximately 36.62% and 17.09% of the variance in the FTIR-ATR spectral data respectively. Fig. 4 b) show the scores of the LDA plotted with LD3 as a third dimension, of which accounted for an additional 13.70% such that LD1, LD2 and LD3 together account for of 67.41% of the variance in the data. The wavelengths with the greatest variation between samples according to the LD1 loading plot were 1720 cm$^{-1}$ and 1015 cm$^{-1}$. Peaks were observed in the spectra of MBCs and hemp at 1015 cm$^{-1}$ and therefore this wavenumber was chosen to calculate average peak ratios to quantify the variation between the specimens.

### Clustering

#### PCA clustering

K-Means clustering was performed on the dataset with the number of clusters set to five. The quality of the clustering was evaluated using the silhouette score, Adjusted Rand Index (ARI), and Normalised Mutual Information (NMI). The silhouette score was approximately 0.447, indicating a moderate level of clustering quality, with values closer to 1 suggesting better-defined clusters. The ARI was calculated to be 0.0769, indicating low agreement between the clusters and the actual specimen groups. The NMI score was found to be 0.2948, reflecting a limited degree of correspondence between the clustering results and the true groupings. These results suggest that while the clustering captures some aspects of the underlying group structure, the alignment with the actual specimen groups is relatively weak. This implies that the current clustering method identifies certain patterns within the data, but may require further refinement or alternative approaches to more accurately reflect the true groupings.

#### LDA clustering

K-means clustering analysis was performed on the LDA results with the number of clusters set to five. The silhouette score for this clustering was approximately 0.514, indicating a moderate level of clustering quality, with higher values suggesting better-defined clusters. The Adjusted Rand Index (ARI) was calculated to be 0.2764, indicating a moderate agreement between the clusters and the actual specimen groups. Furthermore, the Normalised Mutual Information (NMI) score was found to be 0.6671, reflecting a substantial degree of correspondence between the clustering results and the true groupings. These findings suggest that the clustering based on LDA results captures a significant portion of the underlying group structure, demonstrating a better alignment with the actual specimen groups compared to the PCA-based clustering.

### Comparative analysis of peaks

The multivariate analysis found that the wavelength 1640 cm$^{-1}$ contributed largely to PC1 in the PCA conducted on the spectral data from individual specimen, the wavelength 1535 cm$^{-1}$ contributed largely to PC1 in the PCA conducted on the species- averaged spectral data, and the wavelength 1015 cm$^{-1}$ contributed largely to LD1 in the LDA conducted on the spectral data from individual specimen. Therefore % reflectance values at 1640 cm$^{-1}$ ($R_{1640}$), 1535 cm$^{-1}$ ($R_{1535}$) and 1015 cm$^{-1}$ ($R_{1015}$) were used to investigate correlation between FTIR data and thermal conductivity.

**Fig. 6:**
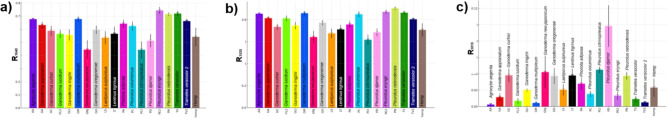
Mean values of $R_{1640}$, $R_{1535}$ and $R_{1015}$ for composites made with different species in this study.

Figure 6 a), b), and c) show the mean values of $R_{1640}$, $R_{1535}$, and $R_{1015}$ respectively for the composites made with each species.

Three one-way ANOVAs were conducted to compare the effect of different groups on the measured values of $R_{1640}$, $R_{1535}$, and $R_{1015}$. The null hypothesis H$_0$ states that there are no significant differences between the group means. The alternative hypothesis H$_1$ states that at least one group mean is different from the others. The results of the analysis are shown in Table 4; the analysis revealed that there is a statistically significant difference between groups for $R_{1640}$, $R_{1535}$, and $R_{1015}$ and in each case H$_0$ is rejected, concluding that not all group means are equal.

**Table 4:** Results of ANOVA tests conducted to compare $R_{1640}$, $R_{1535}$, and $R_{1015}$ between composites made with different species.

	F(18,133)	*p*
$R_{1640}$	4.51	1.46x10-7***
$R_{1535}$	6.75	9.05x10-12***
$R_{1015}$	6.04	1.72x10-10***

**Fig. 7:**
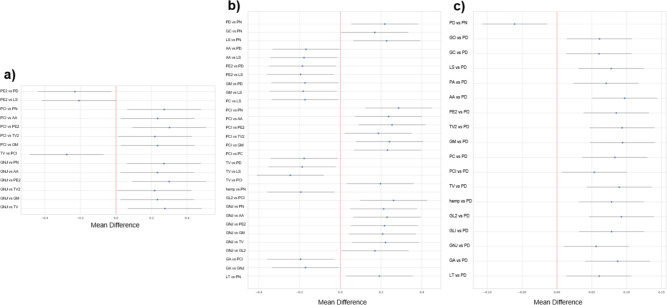
Groups that differed significantly at $p < 0.05$ as tested by a post hoc Tukey test comparing (**a**) $R_{1640}$, (**b**) $R_{1535}$, and $R_{1015}$ measured using FTIR-ATR.

A post hoc Tukey test showed that several groups differed significantly at $p < 0.05$ for $R_{1640}$, $R_{1535}$ and $R_{1015}$. The pairs that differed significantly for each test are shown in Fig. 7.

Comparing $R_{1640}$, *Ganoderma neo-japonicium* and *Pleurotus citrinopileatus* had the most pairwise significant differences. For $R_{1535}$ *Pleurotus citrinopileatus* also had the most pairwise significant differences. For $R_{1015}$ *Pleurotus djamor* had the most pairwise significant differences. Interestingly, the results of the pairwise post hoc Tukey’s tests revealed that species with a common genus are not statistically less likely to show pairwise significant differences in $R_{1640}$, $R_{1535}$, or $R_{1015}$, however the commercial and wild cultures of *Trametes versicolor* did not display significant differences. This was concluded via calculation of a Chi-squared statistic and p- value to test the null hypothesis, $H_0$ that there is no difference in the number of pairwise differences between species from the same genus and species from different genera, conducted at the 0.05 significance level. The p-value obtained for the post hoc Tukey’s test for $R_{1640}$, $R_{1535}$, and $R_{1015}$ was 0.25, 0.16, and 0.17 respectively, therefore we fail to reject $H_0$.

### Analysis of thermal conductivity and FTIR spectra

The relationship between the FTIR multivariate analysis (PCA and LDA groupings) and the measured $\lambda$ was assessed. The Pearson correlation coefficient was calculated for the reflectance value at 3 key FTIR peaks identified using PCA ($R_{1015}$, $R_{1535}$, and $R_{1640}$). Weak correlations were observed ($r=-0.20$, $r=0.15$, $r=-0.01$ respectively), none of which were statistically significant ($p> 0.05$). This indicates that the intensity values at these wavenumbers do not exhibit a meaningful correlation with measured thermal conductivity. Additionally, p-values were calculated for the correlation between all wavelengths in the FTIR-ATR spectra, revealing no statistically significant relationship with thermal conductivity for any wavelength.

Similarly, the correlation between the first two linear discriminant components (LD1 and LD2) and thermal conductivity was examined, yielding values of $r=0.05$ and $r=0.135$, respectively. As these correlations were also not statistically significant ($p> 0.05$), it can be concluded that the positioning of specimens in LDA space does not strongly correspond to their thermal properties.

Overall, the results indicate that the chemical characterisation of the specimens through FTIR does not show a meaningful relationship with the measured thermal conductivity. While chemical changes- potentially arising from variations in fungal digestion of the hemp-shiv-may influence thermal conductivity to some extent, the combination of FTIR-ATR and thermal conductivity measurements appears to lack the sensitivity needed to detect such effects. Instead, other physical factors likely play a more dominant role in the observed variations in thermal conductivity between specimens.

However, the use of multivariate methods such as PCA and LDA, is a valuable approach for analysing large FTIR datasets and identifying differences between specimens. These techniques may be particularly effective in revealing correlations with thermal properties when applied to materials with more pronounced chemical differences, such as those derived from different substrates or subjected to chemical treatments. Additionally, these methods hold value in the classification of MBCs and other bio-based materials, which could be useful in waste management. As MBC production expands to include a wide range of substrates and fungal species, waste MBCs may comprise numerous substrate-species combinations, each potentially requiring different processing strategies. In this context, robust classification methods like PCA and LDA could play a key role in efficiently identifying and sorting waste materials for appropriate recycling or disposal pathways^51^.

## Discussion

The successful production of mycelium-based composites using 18 different fungal strains under the same protocol using a hemp-shiv substrate demonstrates the versatility of MBCs and the broad compatibility of hemp-shiv as a substrate. Given the large volume of hemp-shiv available as a byproduct of hemp processing^52^, its use as a substrate presents a sustainable and abundant resource for MBC production. This is advantageous as it demonstrates that MBC is not dependent on a single fungal species and successful MBC formation is not highly sensitive to changing species. Production was deemed successful upon the formation of four replicates of MBCs with good integrity with full colonisation. The MBCs produced exhibited variations in colour and aesthetic qualities; for instance, the composites made using *Laetiporus sulphureus* had a distinct orange colour. This is important for design and architectural purposes where aesthetic qualities are key factors for material selection, as well as for public acceptance of mycelium-based composites where materials which resemble traditional materials (smooth, even colour) are often preferred^53^.

Thermal conductivity measurements confirmed that all MBCs produced in this study had values of $\lambda$ below 0.1 W/mK, making them good insulation materials. The lowest measured value was $\lambda = 0.0376 \pm 0.0006$ W/m·K for MBCs made using *Pholiota adiposa*, while the highest was $\lambda = 0.0451 \pm 0.001$ W/m·K for MBCs made using *Lentinus tigrinus*, with statistically significant differences measured between MBCs made using different species. However, these differences were relatively small and it would be presumptive to assume that one species consistently provides the best thermal properties. The growth of a given species will be influenced by multiple factors, including the substrate used, growth conditions, and the age and health of the mycelium, thus the ranking of MBCs made using different species is not necessarily fixed. This underscores that while species and strain selection do affect material properties, a single species is not necessarily superior in all situations. Moreover, the relatively small variations in $\lambda$ across different species suggests that, for species capable of successfully colonising and binding a given substrate, overall performance remains largely consistent between different choice of species. This is beneficial when considering production sensitivities, as it suggests a degree of flexibility in species selection without drastically impacting thermal properties. A statistically significant positive correlation between $\lambda$ and $\rho$ was observed. This aligns with the expectation that as density increases porosity of a material decreases, which thereby reduces the proportion of insulating air pockets and increases the proportion of the solid phase responsible for increased heat transfer. The difference in density between the MBCs may be attributable to differences in mycelial growth rates. Changing density, by changing the colonisation time or packing density of the substrate, may be an effective strategy in further reducing $\lambda$, and indeed light-weight insulation materials are preferable for reducing structural load and ease of handling. However, this relationship between $\lambda$ and $\rho$ may not apply beyond the range of densities measured in this study. For lower-density materials, a loss of structural integrity could become a limiting factor, potentially compromising viability and preventing further reductions in $\lambda$.

The MBCs made using different species showed differences in their FTIR spectra, including at key peak wavelengths identified using PCA. These differences may reflect differences in the fungal digestion processes between the different species used which lead to differences in the substrate’s chemical composition. The species used in this study were all selective white rot fungi, as these have demonstrated the greatest success in MBC formation^18^. The differences in how various fungi modify hemp-shiv would likely be more pronounced if species with different digestion modes were included. The selective white rot fungi, to which the species used in this study belong, preferentially degrade lignin while only partially breaking down cellulose and hemicelluloses. This selective degradation helps preserve structural integrity^23^. Other classifications of fungi include brown rot fungi, which primarily break down cellulose and hemicelluloses while leaving behind modified lignin, and general white rot fungi which degrade all major components of lignocellulose^54^. However, including a broader diversity of fungi would pose additional challenges for successful MBC formation, as this is not conducive to substrate binding and structural integrity due to the degradation of structural elements like cellulose and hemicelluloses. Despite the variations observed, no significant correlation was found between the FTIR spectral data and $\lambda$, indicating that chemical changes alone are insufficient to explain the differences in the measured heat transfer properties. This is likely because the digestion mechanisms among tested species were not distinct enough to produce substantial differences in thermal behaviour, with other factors such as density and surface texture possibly being more important in determining $\lambda$. Furthermore, FTIR has certain limitations in this context. While it is a valuable tool for detecting bulk chemical changes, the method used here does not provide information on the spatial distribution of chemical modifications. Additionally, multiple chemical compounds may contribute to the presence or variation of a peak, making subtle changes difficult to detect or quantify.

However, FTIR remains a useful technique for categorising MBCs and tracking substrate modifications. Combining FTIR-ATR with multivariate analysis, as demonstrated in this study, could have future applications in the waste management of bio-based materials by offering a simple method for identifying such materials, such as after their use as insulation, allowing for appropriate waste management strategies to be implemented. Similar approaches have been used to classify natural fibres in buildings^55,56^.

To further explore species-dependent variation and extract meaningful information from the complex spectral data, PCA and LDA were used. PCA was used to identify key wavelengths that explained differences in spectra, with 1640 cm$^{-1}$, 1015 cm$^{-1}$ and 1535 cm$^{-1}$ being wavelengths that contributed to differences between specimens, of which were statistically significant. LDA provided a means to statistically differentiate species based on their FTIR spectra, clustering MBCs based on this. However, while MBCs made using different species can be distinguished based on spectral data, there was no statistically significant relationship between LDA groupings and $\lambda$, again suggesting that other factors are more dominant in determining $\lambda$ in this study.

## Conclusion

This study presents a systematic comparison of mycelium-based composites produced using 18 fungal strains on a hemp-shiv substrate. While species selection influenced composite appearance, chemical signatures, and thermal performance, all species produced materials with thermal conductivities suitable for insulation applications. Chemical differences identified through FTIR-ATR did not correspond to significant differences in thermal conductivity, indicating that physical structure plays a more dominant role in heat transfer than species-specific biochemical modifications. Overall, the results highlight both the versatility of MBCs across fungal species and the importance of physical properties in determining thermal behaviour, whilst demonstrating a method of classifying different types of MBCs using multivariate analysis. The key conclusions are as follows:

- MBCs can be successfully formed from a wide range of fungal species, with 17 species used with the same substrate under the same conditions, demonstrating the versatility of MBCs and supporting the practical flexibility of MBC production.
- Thermal conductivity varied significantly between MBCs formed with different species, but all MBCs achieved values below 0.1 W/m·K, confirming their suitability as sustainable insulation materials.
- Density showed a statistically significant positive correlation with thermal conductivity, suggesting that physical properties may drive thermal property.
- FTIR-ATR revealed chemical differences between MBCs produced using different species, and multivariate analysis (PCA, LDA) was able to classify MBCs into distinct spectral clusters.
- No significant correlation was found between FTIR-ATR determined chemical differences and thermal conductivity, indicating that chemical modifications arising from mycelium digestion do not strongly influence heat transfer under the conditions tested.
- The findings emphasise that optimisation of the thermal properties of MBC insulation materials should prioritise physical properties. Choice of species can, however, affect aesthetic properties.

Future research should investigate how adjustments to growth conditions and substrate properties can further enhance the insulating performance of MBCs, and whether additional chemical or enzymatic treatments could introduce more substantial variations in thermal behaviour. Furthermore, applying PCA and LDA to larger datasets of MBC spectra from diverse species-substrate combinations could improve classification and reveal correlations with other material properties. This study highlights the adaptability of MBCs to different fungal species and provides direction for optimising their overall material properties. By integrating species selection with changing physical properties of the material, MBCs can be further refined as sustainable, low-carbon alternatives to conventional insulation in the construction industry.

## Data Availability

The datasets generated during and/or analysed during the current study are available from the corresponding author on reasonable request.
